# Irisin and adropin decrease in preobese or obese individuals with nonalcoholic fatty liver disease

**DOI:** 10.55730/1300-0144.6110

**Published:** 2025-10-03

**Authors:** Cuma MERTOĞLU, Yaprak Şule ÖREK, Bülent ALBAYRAK, Yusuf Kemal ARSLAN, Abdulkadir ÇOBAN

**Affiliations:** 1 Department of Medical Biochemistry, Faculty of Medicine, İnönü University, Malatya, Turkiye; 2 Department of Medical Biochemistry, Faculty of Medicine, Erzincan Binali Yıldırım University, Erzincan, Turkiye; 3 Department of Internal Diseases, Faculty of Medicine, Atatürk University, Erzurum, Turkiye; 4 Department of Biostatistics, Faculty of Medicine, Erzincan Binali Yıldırım University, Erzincan, Turkiye; 5 Department of Biostatistics, Faculty of Medicine, Çukurova University, Adana, Turkiye

**Keywords:** Nonalcoholic fatty liver disease, adropin, irisin, hematological parameters, atherogenic index

## Abstract

**Background/aim:**

Nonalcoholic fatty liver disease (NAFLD) is a metabolic disease that is commonly observed in individuals with obesity. The present study investigates the irisin and adropin levels of sera in preobese or obese adults with NAFLD.

**Materials and methods:**

Included in the study were 89 patients who were categorized into four groups: Group 1: 25 normal-weight individuals without NAFLD (Control group); Group 2: 17 preobese or obese individuals without NAFLD; Group 3: 24 preobese or obese individuals with Grade 1 NAFLD; and Group 4: 23 preobese or obese individuals with Grade 2–3 NAFLD. The demographic details of all participants were recorded. Abdominal ultrasonography and anthropometric assessments were conducted. Serum adropin and irisin levels were determined using the Enzyme-Linked Immunosorbent Assay (ELISA) method.

**Results:**

Serum adropin and irisin levels were lower in Groups 3 and 4 than in Group 1 (between the all groups; p = 0.006, p = 0.001, respectively), but were comparable between Groups 3 and 4. Furthermore, the atherogenic index of Group 1 was lower than that of Group 4 (p < 0.001). Among the hemogram parameters, the red cell distribution width value was higher in Group 3 than in Group 1 (p = 0.031).

**Conclusion:**

Serum adropin and irisin levels decrease in the presence of NAFLD, regardless of disease severity, which may play a role in the development and exacerbation of NAFLD in preobese and obese individuals.

## 1. Introduction

Nonalcoholic fatty liver disease (NAFLD) is diagnosed based on the detection of hepatic steatosis in radiological images or histological samples, after excluding such secondary causes as alcohol consumption, drug use, or other diseases [1]. Hepatic steatosis is characterized by intrahepatic fat content greater than 5% of liver weight [2]. NAFLD can be divided into two categories based on histological findings: 1) nonalcoholic steatohepatitis (NASH), in cases with steatosis accompanied by inflammation; and nonalcoholic fatty liver (NAFL) if only steatosis is present. In cases with NASH, fibrosis may be present and could advance to cirrhosis [1]. Liver biopsy is the most reliable method for the diagnosis and staging of NAFLD, and is currently the only direct diagnostic approach, however, it is an invasive procedure. This has led to noninvasive methods being investigated for the diagnosis and staging of the disease before NAFLD develops or the severity of the disease increases [3].

Irisin is a peptide that facilitates energy consumption in rats by promoting the transformation of white adipose tissue to brown adipose tissue. Irisin, secreted from muscles after exercise, increases in plasma in both humans and rats. Furthermore, it has been shown to augment energy consumption, decrease body weight, and alleviate insulin resistance (IR) in rats [4].

Adropin, encoded by the ENHO (Energy Homeostasis-Associated) gene, is found in brain and liver tissues[5]. It has been proposed that adropin protects against obesity-related hyperinsulinemia and NAFLD in rats by modulating lipid and glucose metabolism [6].

A review of the literature revealed no studies to date examining the relationship between NAFLD and adropin and irisin levels in individuals with obesity. In the present study, adropin and irisin levels were measured in preobese and obese adults to ascertain their roles in NAFLD pathophysiology, and their relationship with hepatosteatosis was analyzed.

## 2. Materials and methods

### 2.1. Study subjects

This study was carried out with the approval of the Ethics Committee for Clinical Research of Erzincan Binali Yıldırım University Faculty of Medicine on November 27, 2018, with protocol number 34/08. Included in the study were 89 people who applied to the gastroenterology outpatient clinic of a regional training and research hospital between March 25 and June 18, 2019.

Group 1: 25 (12 Female; 13 Male) normal-weight individuals without NAFLD (Control group)
Group 2: 17 (9 Female; 8 Male) preobese or obese individuals without NAFLD
Group 3: 24 (13 Female; 11 Male) preobese or obese individuals with Grade 1 NAFLD
Group 4: 23 (14 Female; 9 Male) preobese or obese individuals with Grade 2–3 NAFLD.

The body mass index (BMI) of the participants was calculated, and classified as follows^1^:

Normal weight: 18.5–24.9 kg/m^2^
Preobese: 25–29.9 kg/m^2^
Obese: > 30 kg/m^2^

Patients who presented to the gastroenterology polyclinic and who were subsequently diagnosed with NAFLD by abdominal ultrasonography (USG) were included in the study. All subjects with NAFLD were diagnosed based on clinical assessment and laboratory evaluations after other liver conditions were excluded. USG imaging was performed by a specialist in the Department of Radiology. The stage of liver steatosis was assessed as Grade I (mild), Grade II (moderate), and Grade III (severe) [7]. The sex, age, weight, height, waist, and hip circumference measurements of the participants were recorded, as well as any tobacco, alcohol, or drug use, presence or history of chronic diseases, medications used, pregnancy or lactation status, hematological disease, and inflammation status. The control group was drawn from individuals who applied to the outpatient clinic for control purposes.

Verbal and written consent was obtained from all those who participated in the study and provided samples for blood analysis, in accordance with the terms of the Declaration of Helsinki. The participants were informed that they were free to leave the study at any time they wanted, and that their access to health services would not be disrupted.

### 2.2. Biochemical analysis

Hemogram, blood glucose, uric acid, blood urea nitrogen (BUN), creatinine, estimated glomerular filtration rate (eGFR), albumin, total protein, alanine aminotransaminase (ALT), aspartate aminotransferase (AST), total and direct bilirubin, gamma-glutamyltransferase (GGT), alkaline phosphatase (ALP), amylase, lipase, serum iron, serum iron binding capacity, creatine kinase (CK), and lactate dehydrogenase (LDH) were recorded, and lipid parameters [low-density lipoprotein (LDL), high-density lipoprotein (HDL), triglyceride (TG), and total cholesterol (TC)] were studied. The atherogenic index was calculated as the logarithm of the ratio of TG to HDL (log(TG/HDL)), and the fibrosis-4 (FIB-4) index was calculated using the formula:


$$\left(\frac{\text{age}\times \text{AST level}}{\text{platelet count}\times \sqrt{\text{ALT level}}}\right).$$

Blood samples were taken from the antecubital vein into a serum separator tube with a gel, and an ethylenediaminetetraacetic acid (EDTA) whole blood tube between 08:00 a.m. and 12:00 p.m. After coagulation, sera were collected by centrifugation at 4000 g for 15 min, and the previously stated biochemical parameters were analyzed. The serum was separated into sample separation tubes for the measurement of adropin and irisin hormones, and transferred to an Eppendorf tube and stored at −80 °C until the study was conducted. Hemogram parameters and HbA1c were analyzed in whole blood.

Serum adropin levels were assessed using an adropin enzyme-linked immunosorbent assay (ELISA) kit (Catalog No: SEN251Hu, USCN) with a stated sensitivity of 12.9 pg/mL, a measurement range of 31.2–2000 pg/mL, and a coefficient of variation (CV) of <10%.

Serum irisin levels were assessed using an FNDC5 ELISA kit (Catalog No: SEN576Hu, USCN). The irisin kit used had a stated sensitivity of 6.9 pg/mL, a measurement range of 15.6–1000 pg/mL, and a CV of <10.

### 2.3. Anthropometric measurements

The participants were weighed on a scale placed on a hard, flat surface after first removing their clothes and shoes, and with their body weight evenly distributed on both feet. For the height measurements, the participants were positioned according to the Frankfurt plane.

Waist circumference measurements were made using a rigid tape measure, with the participant in an standing position, with their hands and arms to their sides and their feet close together. The measurement was taken at the midpoint between the lowermost rib and the beginning of the iliac crest.

The hip measurement was taken with the participant standing upright, and the Frankfurt plane ensured. The highest point of the hip was measured using a tape measure.

The body mass index (kg/m^2^), waist/hip ratio (WHR), and waist/height ratio (WHtR) of the participants were calculated using the obtained measurements.

### 2.4. Statistical analysis

While summarizing the data, descriptive statistics for continuous variables were presented as mean ± standard deviation (SD), median, maximum, and minimum values, while categorical variables were presented as n (%). The normality of continuous variables was evaluated with the Shapiro–Wilk test. In-group comparisons were conducted using ANOVA for variables that met the assumption of normality, followed by post-hoc Bonferroni tests. For variables that did not meet the assumption of normality, the Kruskal–Wallis test was utilized, followed by the post-hoc Dunn’s test. For the examination of relationships between continuous variables, a correlation analysis was conducted, and Pearson or Spearman correlation coefficients were presented based on the distribution type. The ordinal logistic regression analysis considered variables that were significant in the univariate analyses. The group variable (Groups 1, 2, 3, and 4) was utilized as the dependent variable. For the regression analysis, the proportionality of odds was evaluated, and the assumption was found to be met. Variables that could be potential risk factors for liver fat accumulation were identified through regression analysis, and interpretations were made based on the odds ratio (OR) values concerning liver fat. In the evaluations, cases with p ≤ 0.05 were regarded as significant. IBM SPSS Statistics for Windows, Version 22.0 (IBM Corp., 2011) was utilized for the data analysis.

### 2.5. Exclusion criteria

Excluded from the study were patients with liver disorders, chronic inflammation, autoimmune conditions, acute or chronic kidney impairment, hematological disorders, viral infections, malignant tumors, adrenal insufficiency, thyroid dysfunction, type diabetes mellitus (DM), pregnant and lactating women, those taking medications for hypertension and dyslipidemia, alcohol, tobacco, and drug users, and those leading a sedentary lifestyle.

## 3. Results

The clinical information and biochemical results of the four groups are presented in Tables 1 and 2. The mean age of Groups 3 and 4 was higher than that of Groups 1 and 2, and the mean age of Group 2 was higher than that of Group 1. There was no age difference between the participants of Groups 3 and 4 (Table 1).

The waist circumference of Groups 3 and 4 was higher than that of Group 2. The weight, WHtR, WHR, and BMI of Group 4 were higher than those of Group 2. No notable difference in height was observed among the groups (Table 1).

eGFR, ALT, uric acid, GGT, and direct bilirubin differed between the groups (p < 0.001, p = 0.019, p = 0.007, p = 0.022, p = 0.023, respectively). eGFR was lower in Groups 3 and 4 than in Group 1 (p = 0.004, p < 0.001, respectively). ALT levels in Group 4 were higher than those in Group 1 (p = 0.016), and GGT levels were higher than those in Group 2 (p = 0.050). Similarly, uric acid levels in Group 4 were higher than in Group 3 (p = 0.006). Conversely, serum direct bilirubin levels were lower in Group 3 than in Group 1 (0.017). FIB-4 score was lower in Group 1 than in groups 3 and 4 (p = 0.004, p = 0.009 respectively). No other differences were noted between the groups’ biochemical parameters (Table 2).

In the examination of lipid parameters, the HDL levels of Group 4 were lower than those of Group 1, and the atherogenic index of Group 1 was lower than that of Group 4 (p = 0.016, p < 0.001 respectively). Additionally, TG levels in Groups 3 and 4 were higher than in Group 1 (p = 0.030, p < 0.001). Serum cholesterol and LDL levels were higher in Groups 2, 3, and 4 than in the control group (Group 1), although the difference was not significant (Table 3).

**Figure 1:**
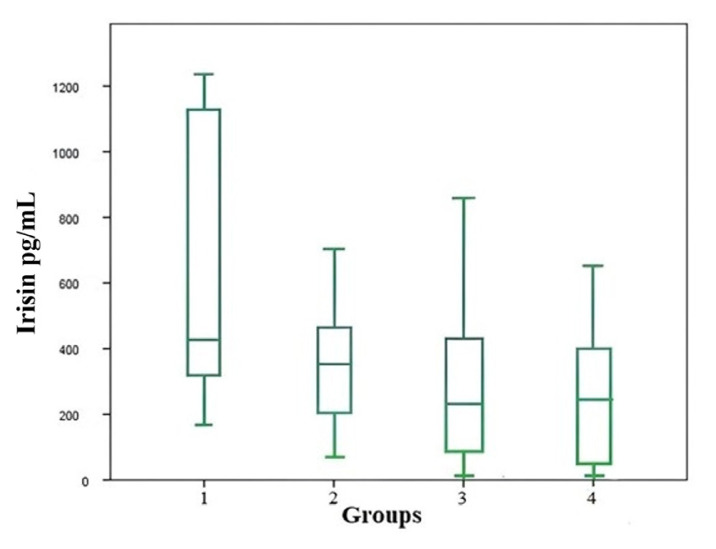
Comparison of serum irisin levels between groups.

**Figure 2:**
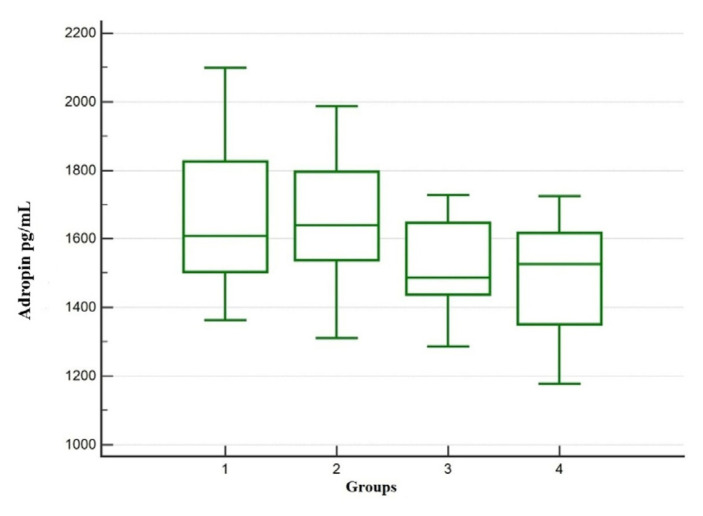
Comparison of serum adropin levels between groups.

Serum adropin and irisin levels were different among the groups (p = 0.006, p = 0.001), being lower in Groups 3 and 4 than in Group 1 (Table 4, Figures 1 and 2)

An analysis of hemogram parameters revealed the red cell distribution width (RDW) value to be higher in Group 3 than in Group 1 (p = 0.031). No other between-group differences were noted in any other hemogram parameters (Table 5).

An analysis of the correlation of the adropin hormone with various parameters revealed a negative correlation with age (r = −0.243, p = 0.038), weight (r = −0.340, p = 0.003), hip circumference (r = −0.364, p = 0.002), waist circumference (r = −0.357, p = 0.002), WHtR (r = −0.342, p = 0.004), BMI (r = −0.375, p = 0.001), glucose ( r = −0.372, p = 0.002), and LDL (r = −0.469, p = 0.009). Specifically, a moderate correlation was noted between LDL and adropin levels. No correlation relationship was found among the remaining variables (Tables 6 and 7).

Similarly, a negative correlation was noted between irisin and age (r = −0.378, p < 0.001), weight (r = −0.347, p = 0.001), hip circumference (r = −0.279, p = 0.011), waist circumference (r = −0.415, p < 0.001), WHR (r = −0.434, p < 0.001), WHtR (r = −0.391, p < 0.001), BMI (r = 0.321, p = 0.003), and atherogenic index (r = −0.341, p = 0.042). Notably, a moderate negative correlation was found between WHR and waist circumference ratio. No correlational relationship was identified among the remaining variables (Tables 6 and 7).

Ordinal logistic regression analysis was conducted to assess the increasing trend in the disease course and severity from Group 3 to Group 4. The analysis revealed that the odds ratios (OR) for adropin and irisin were below 1, and while the odds ratios for BMI, age, ALT, and AST were above 1, only BMI was significant (Table 8).

## 4. Discussion

NAFLD is a significant clinical problem due to the challenges to diagnosis, complex pathogenesis, and the lack of validated treatments [2]. Biopsy remains the optimum approach to the diagnosis of NAFLD; however, its invasive nature makes it challenging for clinical implementation, especially in the pediatric population. While USG and magnetic resonance imaging (MRI) are commonly used for the diagnosis of NAFLD, they come with certain limitations as screening tools, notably their high costs, especially for developing countries. This has prompted research into noninvasive and simple approaches to the diagnosis and staging of the disease before NAFLD develops or the severity of the disease increases, aiming to reduce the need for such invasive methods as liver biopsy [3].

Of particular note in the present study was the decreased levels of irisin in obese patients with NAFLD, which corroborate the findings of previous important studies. In a study conducted by Polyzos et al., lower irisin levels were noted in the obese controls and the NAFL, and NASH patient groups than in the lean patient group [8]. In a study of obese adults who were categorized based on intrahepatic lipid levels, irisin levels were noted to gradually decrease as intrahepatic lipid levels increased. In the same study, individuals with a fatty liver had lower irisin levels than those without. All these studies point to a potentially protective role of irisin in NAFLD. In the present study, however, no statistical significance was noted between Groups 2 and 3, and Groups 2 and 4. This finding warrants further investigation in a larger study to produce more robust evidence, since Group 2 comprised obese individuals without NAFLD, while Groups 3 and 4 included obese individuals with different NAFLD grades. Additionally, an inverse correlation among sera ALT and AST levels and irisin was shown in a previous study [9]. In another recent study, a decrease in irisin was noted in patients with NAFL, type 2 diabetes mellitus (T2DM), and those with both NAFL and T2DM, in a comparison with a control group, and a negative correlation between irisin and visceral adiposity was reported [10]. In the present study the FIB-4 score was higher in the NAFLD patients than in the healthy non-obese individuals, while no significant difference was noted between the healthy non-obese and obese healthy individuals. In the present study, the mean FIB-4 score of the NAFLD patients was lower than the recommended cut-off (1.3) [11,12].

There is much evidence suggesting a decrease in irisin in the sera of individuals with metabolic diseases [13,14], highlighting irisin as a potential therapy target. Irisin, by increasing uncoupling protein 1, can stimulate lipid mobilization and glucose uptake, induce the differentiation of white adipocytes into beige adipocytes, and improve adipose tissue capacity [14]. An earlier study reported a negative correlation between BMI and irisin [10,15], concurring with the present study, although there have also been studies reporting a positive correlation between the two [16,17]. This disparity could be attributed to the different populations studied in the respective investigations. Indeed, the studies reporting a negative correlation were conducted with obese [15] and NAFL [10] groups, while those reporting a positive correlation involved healthy and diabetic groups [16,17].

Although the levels of irisin in the healthy control and healthy obese groups were alike, the identification of a negative correlation among irisin levels and weight, hip circumference, waist circumference, WHR, WHtR, and BMI, suggests a potential role of irisin in central obesity.

Another significant observation from the present study was the decrease in adropin levels in obese patients with NAFLD. In a pediatric study, serum adropin levels were found to be decreased in obese children when compared to the nonobese group, and children with NAFLD had lower adropin levels than the healthy obese group [18]. In a study involving adults, patients with moderate-severe hepatosteatosis had lower adropin levels than the healthy group, and the same study reported a negative correlation between adropin and GGT, TC, and TG [19] In the present study, a negative correlation was identified between LDL and adropin.

In a study conducted on rats, it was suggested that adropin may have effects on glucose and lipid metabolism [5]. Similar to irisin, reduced adropin levels have been linked to such metabolic conditions as T2DM, IR [20], hepatic steatosis [18], polycystic ovary syndrome [21], metabolic syndrome [22], and obesity [23]. Furthermore, in the present study, a negative correlation was noted between adropin levels and hip circumference, waist circumference, WHtR, and WHR. All of these findings suggest a relationship between adropin levels and abdominal obesity.

The results of the ordinal logistic regression analysis conducted in the present study reveal that adropin and irisin may have a protective effect against NAFLD. An increase in irisin levels associated with a 1.006-fold increase in protection against progression to a higher NAFLD stage (from Group 3 to Group 4, in increasing severity), while adropin was found to be 1.007 times more protective. The magnitude of these effects, however, indicates limited clinical significance, and so further studies are recommended to determine whether these modest statistical associations can lead to clinically meaningful outcomes. In addition, a 2-unit increase in BMI was noted to increase the risk/severity of obesity and adiposity 1.6 times, while an approximate 1-unit increase in ALT, AST, and age values was seen to increase the risk/severity of obesity and adiposity.

Fallo et al. reported a strong positive correlation between liver fat content and waist circumference in preobese and obese nondiabetic individuals with NAFLD. Waist circumference measurement increases in NAFLD, and serves as a determinant for the disease [24]. A link has been identified between larger waist circumferences and BMI values and an increased risk of NAFLD [25]. In the same study, waist circumference in all groups was higher than in the healthy and normal-weight control group. These results support the notion that waist circumference may serve as an early indicator of both abdominal obesity and steatosis. It was further noted that the mild to moderate-severe hepatosteatosis groups (Groups 3 and 4, respectively) had a greater waist circumference than the preobese group or obese group without NAFLD. It can be concluded from these results that waist circumference may potentially serve as an indicator of liver fat accumulation, independently of the obesity factor, and can be utilized as an easily applicable approach, although further comprehensive studies are needed to confirm this.

The WHtR and WHR ratio of the obese or preobese groups with hepatosteatosis were higher than those of the healthy and normal-weight control group (Group 1). Furthermore, the aforementioned ratios of the moderate–severe hepatosteatosis group (Group 4) were higher than in the non-NAFLD obese group (Group 2). These findings indicate that WHtR and WHR ratios may not increase in the early stages of NAFLD, but increase as the severity of the disease increases. In a recent study a positive regression relationship was determined between WHtR and fatty liver index [26]. While the correlation between WHtR and central obesity has been well researched, studies investigating its relationship with NAFLD have to date been limited. In one recent study, a nonlinear positive regression relationship was reported between WHtR and NAFLD [27].

Previous studies have identified the atherogenicity index as a strong marker of susceptibility to atherosclerosis and coronary heart disease [28, 29]. To the best of our knowledge, however, there have been few studies to date assessing its role in NAFLD. Given the close correlation between TG, HDL, and NAFLD, it can be hypothesized that the atherogenic index may serve as a determinant for NAFLD. In a study of an obese cohort, a relationship was noted between a high atherogenicity index and BMI, waist circumference, ALT, GGT, and lipid profile values. The study thus suggested that the plasma atherogenic index could be utilized as an adjunctive diagnostic measure for NAFLD [30]. In yet another study, it was reported that TC levels [18], LDL [25], and TG [19] tend to increase in the presence of hepatic steatosis, while HDL levels decrease [25]. Consistent with these findings, the group with moderate-severe steatosis (Group 4) in the present study exhibited higher TG and atherogenic index values than the control group (Group 1), along with lower HDL levels.

It has been well established that a link exists between elevated RDW and metabolic syndrome and its components [31]. Yang et al. reported an increase in RDW levels in NAFLD patients and significant associations with WHR, BMI, fasting plasma glucose, and triglycerides (TG) concerning NAFLD risk [32]. In a separate study, high RDW levels were suggested to be associated with progressed fibrosis in NAFLD, and although the pathophysiology of this relationship was not clearly specified, it is believed that common interactions with inflammation and oxidative stress could explain this association [33]. Conversely, in another study, no relationship between RDW levels and NAFLD severity was observed [34]. In the present study, the group with mild hepatic steatosis had higher RDW levels than the healthy controls, while RDW was similar between the groups with mild and moderate-severe hepatic steatosis.

Neutrophil-to-lymphocyte ratio (NLR) and mean platelet volume (MPV) are considered cost-effective and simple inflammation markers in many diseases. A multicentric study revealed increased NLR and MPV levels in patients with NASH, compared to NAFLD patients without NASH. This increase was reported to be more pronounced in cases with advanced fibrosis, while there were no differences in NLR and MPV of the groups [35].

The limitations of the present study include the limited number of patients with hepatosteatosis and the use of USG for diagnosis, given its low accuracy in detecting mild steatosis. Furthermore, the physical activity status of the patients was not considered, which may affect certain parameters, especially irisin concentrations. Measurement methods that provide more detailed information about body fat and body composition (such as bioelectrical impedance analysis) instead of the anthropometric measurements used in the present study may produce more reliable results, and provide a better understanding of the effects of adropin and irisin on fat mass, including regional adiposity.

## 5. Conclusion

Serum irisin and adropin levels decrease in the presence of NAFLD, regardless of disease stage, and this decrease may contribute to the development and exacerbation of NAFLD in preobese and obese individuals. Moreover, the negative correlation of adropin and irisin with central adiposity suggests a potential association of these peptides with visceral adiposity.

## Figures and Tables

**Table 1:** Comparison of demographic characteristics and anthropometric measurements between groups.

Variables	Group 1 n=25	Group 2 n= 17	Group 3 n= 24	Group 4 n= 23	p-values	Between-group comparisons (p-values)
Age (Year) +	27.00±5.7	36.71±9.37	46.79±10.14	45±10.08	<0.001	1–2 (0.005) 1–3, 1–4 (<0.001) 2–3 (0.004) 2–4 (0.028)
Height (m) +	1.68±0.09	1.67±0.08	1.63±0.09	1.65±0.12	0.159	
Weight (kg) +	63.88±10.21	78.35±8.92	81.13±11.35	88.3±10.46	<0.001	1–2, 1–3, 1–4 (<0.001) 2–4 (0.021)
BMI (kg/m^2^) +	22.38±1.97	27.93±2.23	30.96±5.87	33.13±6.75	<0.001	1–2 (0.002) 1–3, 1–4 (<0.001) 2–4 (0.007)
Waist Circumference (cm) + **	80.88±9.36	97.88±5.6	106.4±10.52	111.3±11.88	<0.001	1–2, 1–3, 1–4 (<0.001) 2–3 (0.048) 2–4 (<0.001)
Hip Circumference (cm) *	100 (85–111)	110 (97–120)	111 (97–120)	115 (97–120)	<0.001	1–2 (0.001) 1–3, 1–4 (<0.001)
Waist/Hip Ratio (cm) +	0.81±0.07	0.89±0.05	0.93±0.05	0.94±0.05	<0.001	1–2, 1–3, 1–4 (<0.001) 2–4 (0.048)
Waist/Height Ratio (cm) *	0.48 (0.41–0.58)	0.58 (0.52–0.64)	0.65 (0.55–0.76)	0.68 (0.56–0.92)	<0.001	1–2 (0.003) 1–3, 1–4 (<0.001) 2–4 (0.047)

+ Mean ± SD

* Median, IQR

** p-value for sex comparison: 0.817

(Group 1: 25 normal-weight individuals without NAFLD, Group 2: 17 preobese or obese individuals without NAFLD, Group 3: 24 preobese or obese individuals with NAFLD grade 1, Group 4: 23 preobese or obese individuals with NAFLD grades 2, 3) (BMI: Body mass index)

**Table 2:** Comparison of biochemical parameters between groups.

Variables	Group 1 n=16	Group 2 n=10	Group 3 n=18	Group 4 n=14	p-values	Between-group comparisons (p-values)
Glucose (mg/dL) *	87.93 (72–112)	91 (73–120)	94 (71–162)	95 (76–123)	0.304	
BUN (mg/dL) +	11.76±2.42	13.61±4.76	13.81±3.94	14.52±3.42	0.091	
Creatinine (mg/dL) *	0.77 (0.67–0.98)	0.83 (0.65–3.35)	0.71 (0.56–1)	0.76 (0.55–1.06)	0.259	
eGFR+	115.6±10.44	105.7±12.11	102.9±10.29	99.73±15.1	<0.001	1–3 (0.004) 1–4 (<0.001)
ALT (U/L) *	17 (5.42–75)	20 (10–71)	19 (11–83)	28 (14–141)	0.019	1–4 (0.016)
Albumin (g/L) +	45.82±4.65	44.54±3.42	43.99±3.36	46.21±3.34	0.228	
ALP (U/L) *	78 (59–109)	72 (40–130)	78 (56–165)	82 (55–274)	0.526	
AST (U/L) *	17.32 (10–58)	17 (12–28)	18 (12–43)	22 (15–60)	0.052	
Amylase (U/L) +	56.8±16.72	70.14±19.97	65.95±21.17	58.78±18.34	0.241	
Total protein (g/L) *	75.44 (60.5–81.2)	72.58 (68.42–85.64)	76.6 (65.36–81.6)	77.3 (72.2–85.3)	0.123	
Lipase (U/L) *	19 (12–46)	32 (9–54)	22 (4–73.2)	20 (10.56)	0.281	
Uric acid(mg/dL) *	4.28 (3.26–5.22)	4.25 (2.43–7.3)	3.88 (3.06–7.34)	6.31 (3.95–8.19)	0.007	3–4 (0.006)
Total bilirubin (mg/dL) *	0.67 (0.35–1.47)	0.77 (0.29–1.2)	0.51 (0.17–1.65)	0.86 (0.32–1.81)	0.067	
Direct bilirubin (mg/dL) *	0.28 (0.18–0.85)	0.25 (0.10–0.39)	0.19 (0.10–0.56)	0.28 (0.15–0.46)	0.023	1–3 (0.017)
Indirect bilirubin (mg/dL) *	0.41 (0.06–2.70)	0.50 (0.15–0.81)	0.32 (0.13–1.09)	0.50 (0.17–1.30)	0.568	
GGT (U/L) *	21 (8–67)	18 (8–97)	32 (15–143)	32 (13–327)	0.022	2–4 (0.050)
Serum iron (μg/dL) *	87 (25–149)	71 (45–117)	61 (33–148)	71 (24–118)	0.702	
LDH (U/L) +	175.8±27.2	262.6±217.7	187.7±39.3	192.3±38.79	0.553	
FIB-4 score +	0.46±0.16	0.59±0.34	0.74±0.30	0.73±0.27	0.002	1–3(0.004) 1–4(0.009)

+ Mean ± SD

* Median, IQR

(Group 1: 25 normal-weight individuals without NAFLD, Group 2: 17 preobese or obese individuals without NAFLD, Group 3: 24 preobese or obese individuals with NAFLD grade 1, Group 4: 23 preobese or obese individuals with NAFLD grades 2, 3) (BUN: Blood urea nitrogen, ALT: Alanine aminotransaminase, AST: Aspartate aminotransferase, ALP: Alkaline phosphatase, eGFR: estimated glomerular filtration rate, GGT: Gamma-glutamyltransferase, LDH: Lactate dehydrogenase, **FIB-4: Fibrosis-4 score**)

**Table 3:** Comparison of lipid parameters between groups.

Variables	Group 1 n=16	Group 2 n=10	Group 3 n=18	Group 4 n=14	p-values	Between-group comparisons (p-values)
TC (mg/dL) *	168 (131–215)	215 (138–284)	185 (157–257)	234 (130–257)	0.161	
HDL (mg/dL) *	57.42 (36.8–89.96)	42.16 (7.23–41.01)	49.35 (33–59.77)	41.43 (30.7–66.45)	0.008	1–4 (0.016)
LDL + (mg/dL)	97.49±31.76	121±27.96	130.96±30.94	118.2±32.77	0.164	
TG (mg/dL) *	83 (40–108)	130,5 (71–231)	145 (50–319)	209 (110–307)	<0.001	1–3 (0.030) 1–4 (<0.001)
Atherogenic index (mg/dL) *	1.56 (0.45–2.34)	3.21 (1.51–5.63)	2.78 (0.92–9.67)	5.7 (2.02–7.68)	0.001	1–4 (<0.001)

+ Mean ± SD

* Median, IQR

(Group 1: 25 normal-weight individuals without NAFLD, Group 2: 17 preobese or obese individuals without NAFLD, Group 3: 24 preobese or obese individuals with NAFLD grade 1, Group 4: 23 preobese or obese individuals with NAFLD grades 2, 3) (TC: Total cholesterol, HDL: High density lipoprotein, LDL: Low density lipoprotein, TG: Triglyceride. Atherogenic index: logTG/HDL)

**Table 4:** Comparison of adropin and irisin levels between groups.

Variables	Group 1 n=16	Group 2 n=10	Group 3 n=18	Group 4 n=14	p-values	Between-group comparisons (p values)
Adropin (pg/mL) +	1668±237.4	1649.±182.6	1518.±145.1	1490±169.0	0.006	1–3 (0.031) 1–4 (0.011)
Irisin (pg/mL) *	429.6 (167.1–1233)	354.5 (71.60–1003)	240.9 (12.16–855.80)	243.4 (12.16–650.34)	0.001	1–3 (0.008) 1–4 (0.002)

+ Mean ± SD

* Median, IQR

(Group 1: 25 normal-weight individuals without NAFLD, Group 2: 17 preobese or obese individuals without NAFLD, Group 3: 24 preobese or obese individuals with NAFLD grade 1, Group 4: 23 preobese or obese individuals with NAFLD grades 2, 3)

**Table. 5:** Comparison of hemogram parameters between groups.

Variables	Group 1 n= 16	Group 2 n= 10	Group 3 n= 18	Group 4 n= 14	p-values
WBC (10^3^/uL)+	7.16±1.78	7.59±1.73	7.63±1.94	7.76±2.2	0.811
RBC (10^6^/uL)+	5.25±0.54	5.25±0.54	5.18±0.44	5.37±0.47	0.432
Hemoglobin (g/dL)+	15.77±2.52	14.83±1.76	14.75±2.05	15.46±186	0.409
Hematocrit (%)+	44.58±3.93	43.83±4.30	43.69±4.72	46.01±4.84	0.309
MCV (fL)*	85.15 (64–92.5)	84.65 (76.7–93)	84.85 (67.4–94.1)	86 (66.2–92)	0.844
MCH (pg)+	29.25±1.61	28.81±1.96	28.46±2.6	28.66±2.5	0.652
MCHC (g/dL)+	34.4±1.14	33.78±1.24	34±1.72	33.51±1.45	0.203
Thrombocyte (10^3^/uL)*	285 (165–385)	271 (222–364)	274 (186–417)	270.5 (171–412)	0.963
RDW (%)*	12.5 (11.6–15.6)	12.7 (11.5–15.8)	13.2¥ (11.5–17.9)	13.1 (11.9–17.8)	0.030
PDW (fL)+	12.27±2.14	12.11±1.64	11.86±1.46	12.09±1.7	0.884
MPV (fL)*	10.25 (8.9–12.7)	10.25 (9.1–11.5)	10.4 (8.9–11.4)	10.2 (8.7–11.5)	0.982
P_LCR (%)+	28.08±8.01	27.87±5.67	26.90±5.65	26.92±6.82	0.906
Plateletcrit (%)*	0.28 (0.19–0.39)	0.27 (0.24–0.44)	0.30 (0.17–0.50)	0.26 (0.15–0.45)	0.771
Neutrophil (10^3^/uL)+	4.25±1.59	4.34±1.48	4.71±1.66	4.37±1.76	0.795
Lymphocyte (10^3^/uL)+	2.16±0.64	2.20±0.77	2.19±0.67	2.54±0.68	0.217
Monocyte (10^3^/uL)*	0.54 (0.31–1.40)	0.64 (0.33–1.19)	0.53 (0.25–1.56)	0.52 (0.06–1.20)	0.595
Eosinophil (10^3^/uL)*	0.095 (0.01–0.29)	0.1 (0.03–0.72)	0.15 (0.00–0.39)	0.11 (0.02–0.75)	0.873
Basophil (10^3^/uL)*	0.05 (0.03–0.09)	0.045 (0.03–0.09)	0.04 (0.01–0.09)	0.05 (0.01–1.0)	0.718
NLR (10^3^/uL)*	1.64 (0.82–5.09)	1.92 (1.04–8.49)	1.91 (1.19–24.73)	1.56 (0.57–3.55)	0.439
PLR (10^3^/uL)*	141.06 (67.91–276.4)	125.64 (74.85–415.2)	121.39 (81.23–612.1)	109.04 (73.25–161.7)	0.225

+ Mean ± SD

* Median, IQR

¥ Different from Group 1, p = 0.031)

(Group 1: 25 normal-weight individuals without NAFLD, Group 2: 17 preobese or obese individuals without NAFLD, Group 3: 24 preobese or obese individuals with NAFLD grade 1, Group 4: 23 preobese or obese individuals with NAFLD grades 2, 3) (MCV: Mean corpuscular volume, MCH: Mean corpuscular hemoglobin MHCH: Mean corpuscular hemoglobin concentration, RDW: Red blood cell distribution width, PDW: Platelet distribution width, MPV: Mean platelet volume, P-LCR: Platelet large cell ratio, NLR: Neutrophil to lymphocyte ratio, PLR: Platelet to lymphocyte ratio)

**Table 6:** Comparisons of the correlation relationship between demographic parameters and adropin and irisin.

Parameters		Adropin	Irisin
**Age**	r	−0.243	−0.378
	p	0.038	<0.001
**Weight**	r	−0.340	−0.347
	p	0.003	0.001
**Height**	r	0.130	0.484
	p	0.274	0.078
**BMI**	r	−0.375	−0.321
	p	0.001	0.003
**Waist circumference**	r	−0.357	−0.415
	p	0.002	<0.001
**Hip circumference**	r	−0.364	−0.279
	p	0.002	0.011
**Waist/hip ratio**	r	−0.222	−0.434
	p	0.060	<0.001
**Waist/height ratio**	r	−0.342	−0.391
	p	0.004	<0.001

(BMI: Body mass index)

**Table 7:** Correlation of biochemical parameters with adropin and irisin.

Parameters		Adropin	Irisin
**Glucose**	r	−0.372	−0.091
	p	0.002	0.456
**ALT**	r	0.142	−0198
	p	0.205	0.077
**Albumin**	r	0.207	0.048
	p	0.096	0.704
**ALP**	r	−0.02	−0.001
	p	0.871	0.996
**AST**	r	0.219	−0.182
	p	0.053	0.109
**GGT**	r	0.051	−0.18
	p	0.686	0.145
**LDH**	r	−0.135	0.04
	p	0.54	0.988
**Total cholesterol**	r	−0.164	−0.142
	p	0.336	0.382
**HDL**	r	−0.077	0.213
	p	0.66	0.194
**LDL**	r	−0.469	0.008
	p	0.009	0.963
**TG**	r	−0.015	−0.282
	p	0.933	0.082
**Atherogenic index**	r	0.001	−0.341
	p	0.995	0.042
**Adropin**	r		0.03
	p		0.098
**Irisin**	r	0.03	
	p	0.098	

(ALT: Alanine aminotransaminase, AST: aspartate aminotransferase, ALP: Alkaline phosphatase, Gamma-glutamyltransferase, LDH: Lactate dehydrogenase, HDL: High density lipoprotein, LDL: Low density lipoprotein, TG: Triglyceride, Atherogenic index: logTG/HDL).

**Table. 8:** Ordinal logistic regression analysis, OR for nonalcoholic fatty liver disease.

	Wald	Sig	OR	Lower	Upper
**BMI**	16.278	<0.001	1.622	1.282	2.051
**Adropin**	9.302	0.002	0.993	0.988	0.997
**Irisin**	9.4	0.002	0.994	0.991	0.998
**Age**	0.01	0.921	1.004	0.928	1.086
**ALT**	1.535	0.215	1.031	0.982	1.082
**AST**	0.637	0.425	1.041	0.944	1.147

(Interpretation made using odds ratio (OR) and 95% confidence intervals. ALT: Alanine aminotransaminase, AST: aspartate aminotransferase, BMI: Body mass index.
